# Relationships Between Adiposity Measures and Heart Rate Variability in Children and Adolescents

**DOI:** 10.1007/s00246-025-03924-3

**Published:** 2025-07-10

**Authors:** William T. Juckett, Nicholas G. Evanoff, Aaron S. Kelly, Eric M. Bomberg, Donald R. Dengel

**Affiliations:** 1 School of Kinesiology, University of Minnesota, Minneapolis, MN USA; 2 Department of Pediatrics, University of Minnesota Medical School, Minneapolis, MN USA; 3 Center for Pediatric Obesity Medicine, University of Minnesota Medical School, Minneapolis, MN USA; 4 Division of Endocrinology, Department of Pediatrics, University of Minnesota Medical School, Minneapolis, MN USA

**Keywords:** Adiposity, Autonomic nervous system, Obesity, Children, Adolescents

## Abstract

Understanding relationships between specific measures of adiposity and heart rate variability (HRV) allows for better understanding obesity as a risk factor for cardiovascular and autonomic dysfunction. We sought to examine associations between specific adiposity depots and cardiovascular autonomic function assessed via HRV among children and adolescents. We performed a secondary cross-sectional analysis of a previously completed study of 8–17-year-olds (n = 325; 12.4 ± 2.7 years). Adiposity was assessed via dual x-ray absorptiometry. Resting HRV measures included root mean square of successive differences (RMSSD), percentage of successive normal-to-normal intervals differing by more than 50 ms (pNN50), low frequency power (LF), high frequency power (HF), and low frequency-to-high frequency ratio (LF:HF). Associations between adiposity and HRV were evaluated using linear regression modeling, adjusting for sex and Tanner stage. RMSSD and pNN50 were both negatively associated with total fat (β = − 0.558, *p* = 0.004; β = − 0.178, *p* = 0.034), percent body fat (β = − 0.790, *p* = 0.007; β = − 0.296, *p* = 0.019), trunk fat (β = − 1.024, *p* = 0.004; β = − 0.344, *p* = 0.022), android fat (β = − 0.005, *p* = 0.006; β = − 0.002, *p* = 0.028), gynoid fat (β = − 0.003, *p* = 0.005; β = − 0.001, *p* = 0.034), visceral adipose tissue (β = − 0.013, *p* = 0.016; β = − 0.005, *p* = 0.038) and subcutaneous abdominal adipose tissue (β = − 0.007, *p* = 0.004; β = − 0.003, *p* = 0.017). RMSSD was negatively associated with appendicular fat (β = − 1.152,* p* = 0.008). Visceral adipose tissue was associated positively with LF and LF: HF (β = 0.004, *p* = 0.028; β = 0.019, *p* = 0.033) and negatively with HF (β = − 0.004, *p* = 0.028). We found that excess total and regional adiposity may have negative effects on cardiovascular autonomic nervous system function in a large cohort of children and adolescents. Longitudinal studies are needed to confirm and determine how these relationships change over time**.**

**Supplementary Information:**

The online version contains supplementary material available at 10.1007/s00246-025-03924-3.

## Introduction

Childhood and adolescent obesity are associated with increased cardiometabolic risk factors and comorbidities, leading to increased risk for chronic diseases including type 2 diabetes mellitus and cardiovascular disease [1–6]. While weight status (e.g., normal weight, overweight, obesity) is generally followed using body mass index (BMI), BMI cannot differentiate between lean and fat mass, nor region specific composition. Precisely measured adiposity, particularly in specific depots (e.g., visceral), may be more impactful to risk and co-morbidity development [7].

The autonomic nervous system (ANS), involving a complex interplay between the sympathetic and parasympathetic branches, plays a major role in maintaining physiologic homeostasis, including the regulation of heart rate and heart rate variability (HRV). While heart rate is the number of beats within a 1-min interval, HRV is the variation in time between heartbeats. Worsened (lesser) HRV has been shown to be a strong predictor of future health problems, and is correlated with higher cardiometabolic risk and all-cause mortality [8–10].

HRV analysis is a non-invasive and commonly used method to measure autonomic nervous system function, specifically cardiovascular autonomic nervous system (cANS). HRV research generally utilizes time domain (variability as a function of time) and/or frequency domain (variability as a function of frequency) measures. Time domain measures result from adaptive changes in HR caused by sympathetic and parasympathetic interaction, with lower time domain HRV measures generally understood to indicate worse autonomic nervous system function [11, 12]. Frequency domain measures are often expressed as low-frequency (LF) and high-frequency (HF) power in normalized units (NU), accounting for changes in total power [11, 12]. LF band reflects baroreflex, and the HF band typically reflects parasympathetic or vagal activity, with reduced HF activity observed in numerous cardiac pathologies [13]. HRV has been shown to be a highly reliable measure of autonomic function when assessed under standardized conditions and in clinical settings [14, 15].

Accumulation of excess adiposity, predominately as white adipose tissue in humans, is known to produce bioactive substances (i.e. adipokines) which can have endocrine actions (e.g., increased insulin resistance), and can induce systemic inflammatory stress [16]. Previous research has shown negative relationships between inflammation and HRV measures, and that the presence of obesity may negatively impact nervous system function in both adults [17, 18] and youth [19–23]. Studies in adults have shown associations between surrogate measures of adiposity and HRV differ between central and overall adiposity, such that central adiposity appears to be negatively associated with HRV while overall adiposity may not be [24].

Few studies have evaluated direct associations between adiposity (e.g., visceral adipose tissue [VAT]) and HRV measures (e.g., root mean squared of successive differences [RMSSD]) in children and adolescents [20, 25–28], and fewer have examined whether central and peripheral adiposity are differentially related to HRV. The previous studies examining adiposity alongside HRV have been limited in their sample size and/or analyses, necessitating more robust analyses with a large sample size to advance our understanding of the differential impacts of adipose tissue depots on HRV.

Sex hormones (estrogen and testosterone), which are mediated by gonadotropins (i.e., luteinizing hormone [LH], follicle stimulating hormone [FSH]), are important facilitators during pubertal development. Estrogen and testosterone have both shown to influence fat distribution in youth populations [29] and may contribute to differential inflammatory responses compared to adulthood, understanding their potential associations with HRV may improve our understanding of early onset cardiovascular risk. Therefore, a secondary aim was to examine associations between gonadotropins, as well as sex hormones, and HRV measures among a cohort of children and adolescents with weight statuses ranging from normal weight to obesity.

## Methods

### Study Design and Participants

This cross-sectional secondary analysis utilized data collected from a previously completed study evaluating potential biomarkers and cardiovascular disease risk factors among 8–17-year-olds with BMIs ranging from normal weight to obesity (NCT01508598) [30, 31]. 382 participants originally completed the study, those without HRV data were excluded from analysis, additionally participants with diagnosed type 2 diabetes mellitus, born premature, or diagnosed with a major cardiac disorder (e.g., cardiomegaly, Von Willebrand disease) were excluded from analysis. The protocol for this parent study was approved by our university’s Institutional Review Board, and written assent and consent were obtained from participants and their parent/guardian(s), respectively, prior to any study procedures. Race/Ethnicity was self-reported and included non-Hispanic White, non-Hispanic Black, Hispanic/Latino, Asian, American Indian/American Native, multi-race, and other. Secondary, sub-analyses examining associations between gonadotropins (LH, FSH) and sex hormones (estradiol, testosterone), and HRV measures, were done using a subset of participants with available measures.

### Anthropometric and Body Composition Measures

Height and weight were measured using a standard calibrated wall-mounted stadiometer and digital scale, respectively. Participants were grouped by BMI into normal weight (5th to < 85th BMI percentile), overweight (85th to < 95th BMI percentile), and obesity (≥ 95th BMI percentile) categories based on U.S. Centers for Disease Control and Prevention (CDC) age- and sex-adjusted BMI percentiles [32, 33].

Body composition, including total fat mass (FM) and percent body fat (%BF), was determined by whole-body dual X-ray absorptiometry (DXA; iDXA, GE Healthcare; enCORE software; platform version 16.2). Automatically created regions of interest were used to measure trunk FM, android FM (area between ribs and pelvis), gynoid FM (hips and upper thigh), and visceral adipose tissue (VAT). VAT was estimated using CoreScan (GE Healthcare) as previously described [34, 35]. Appendicular fat was calculated by subtracting trunk FM from total body FM, and subcutaneous abdominal adipose tissue (SAAT) was calculated by subtracting VAT from android FM.

### Heart Rate Variability Measures

HRV testing was performed in a quiet, temperature-controlled environment (22-23^o^ C) with the participant in the supine position, following a 15-min rest period. Three electrodes were placed on the top of the sternum (inferior to the sternal notch), inferior to the xiphoid process, and lateral to the anterior left superior iliac spine. HRV data was collected via a three-lead electrocardiogram (ECG) system (SphygmoCor MM3, AtCor Medical, Sydney, Australia) for a 5-min period. Following data capture, SphygmoCor MM3 software (AtCor Medical, Sydney, Australia, Software version 8.0) performed spectral analysis for time domain measures including RMSSD and percentage of successive normal-to-normal intervals differing by more than 50 ms (pNN50). Normal-to-normal intervals are intervals between normal heartbeats, excluding non-normal or ectopic beats, and are a frequently used measure of time between heartbeats. Frequency domain measures included low frequency power (LF) power, high frequency (HF) power, and LF:HF ratio.

### Measurements of Gonadotropins, Sex Hormones, and Pubertal Status

Blood sample collection was performed in the morning following a ≥ 8 h fast. LH, FSH, estradiol, and testosterone levels were obtained at a Clinical Laboratory Improvement Amendments (CLIA)-certified laboratory using standard procedures. Chemiluminescent immunoassays were used to measure LH and FSH, while liquid chromatography—tandem mass spectrometry (LCMS-MS) was used to assess total testosterone and estradiol. Pubertal status (Tanner stage) was determined via visual inspection of pubic hair performed by trained practitioners using standard methodology [25]. Participants were stratified into pre-pubertal (Tanner stage 1), pubertal (stages 2–4), and post-pubertal (stage 5) categories.

### Statistical Analysis

Descriptive statistics were quantified as frequency distributions with percentages (%) or means with standard deviations (SDs), as appropriate. Linear regression was used to evaluate associations between body composition and HRV measures, with models adjusted for sex and Tanner stage. Linear regression was also used to evaluate associations between gonadotropins and sex hormones, and HRV measures, with models stratified by sex and adjusted for Tanner stage category. To address the issue of non-normality observed in LF:HF ratio, a natural logarithmic transformation was applied prior to statistical analysis. Because the effect of hormones differs between females and males, stratification by sex was utilized in models involving associations with gonadotropins and sex hormones. All analyses were completed using R Studio (R Foundation for Statistical Computing, Version 2023.06.0 + 421, Vienna, Austria) with a two-sided significance threshold of *p* < 0.05. Visualizations were completed using GraphPad Prism (GraphPad Software, Version 10.4.2, San Diego, California USA).

## Results

### Participant Characteristics

**Fig. 1:**
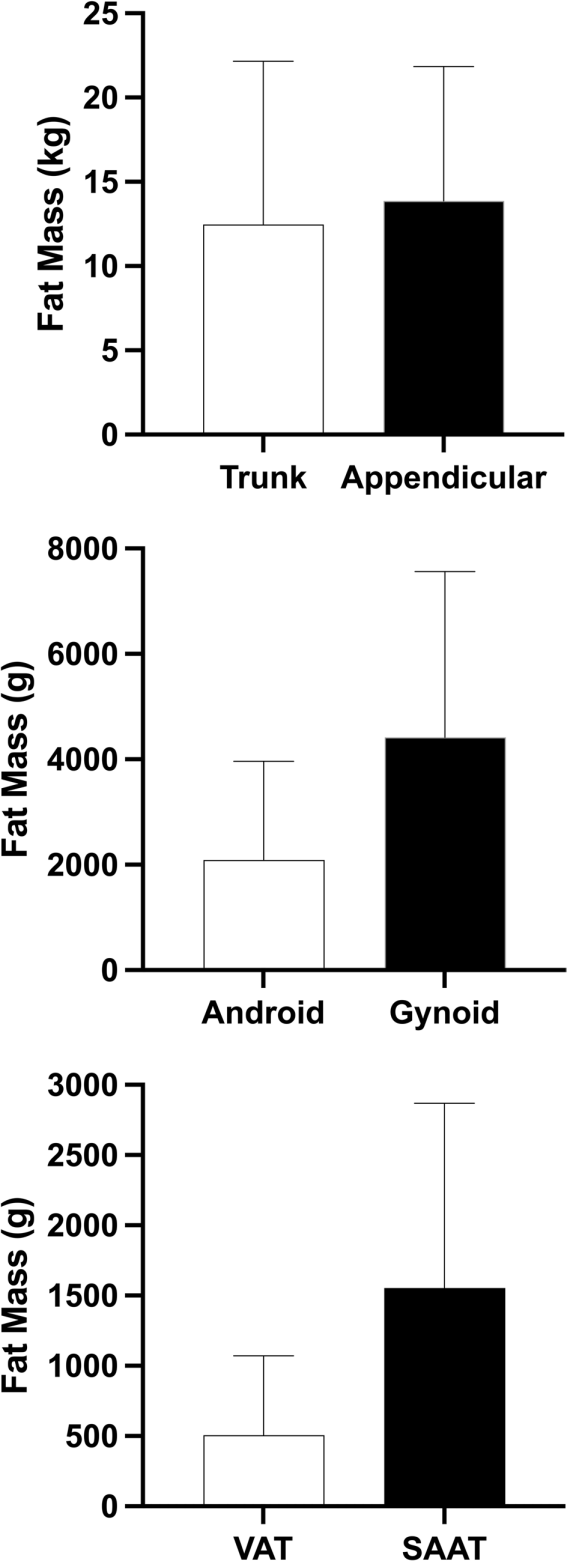
Regional Body Composition measures for the total population. Abbreviations: SAAT, subcutaneous abdominal adipose tissue; VAT, visceral adipose tissue

Table 1 shows baseline characteristics of the 325 children and adolescents (8–17 years) included in our analyses. Of the total population, 52% were female and the majority (57%) were pubertal (Tanner stage 2–4). Participants predominately identified as non-Hispanic White (69%). 55% were categorized as having obesity. Table 2 presents anthropomorphic, body composition, and HRV measures for the total population, and Fig. 1 shows mean ± SD for regional body composition measures (e.g., trunk, appendicular).

**Table 1:** Baseline Characteristics of 325 Children and Adolescents (8–17 years)

Age (years), mean (SD)	12.4 (2.7)
Female, n (%)	168 (52)
Race/Ethnicity, n (%)	
White (non-Hispanic)	224 (69)
Black (non-Hispanic)	31 (11)
Hispanic/Latino	38 (12)
Asian	6 (2)
Multi-Racial	23 (6)
Other	3 (1)
Tanner Stage, n (%)	
Stage I	86 (26)
Stage II	57 (18)
Stage III	57 (18)
Stage IV	67 (21)
Stage V	48 (15)
Weight Status, n (%)	
Normal Weight	124 (38)
Overweight	22 (7)
Obesity	178 (55)

**Table 2:** Mean (SD) Measures of Body Composition and Heart Rate Variability

	**Mean ± SD**
Height (cm)	157.4 (13.9)
Weight (kg)	68.1 (29.1)
Total Fat Mass (kg)	26.34 (17.46)
Percent Fat (%)	35.64 (11.29)
Resting Heart Rate	75.2 (11.0)
HRV Measures	
RMSSD (ms)	89.68 (54.63)
pNN50 (%)	43.89 (23.22)
LF (NU)	41.09 (18.00)
HF (NU)	58.91 (18.00)
LF:HF	0.95 (0.96)

Abbreviations: HF, high frequency; LF, low frequency; LF:HF, low frequency to high frequency ratio; NU, normalized units; pNN50, percentage of NN intervals greater than 50 ms; RMSSD, root mean square of successive differences

### Relationship of Heart Rate Variability and Body Composition

Table 3 shows results from linear regression models evaluating associations between total body adiposity and HRV measures, adjusted for sex and Tanner stage. RMSSD (− 0.558 ms/kg, *p* = 0.004) and pNN50 (− 0.178%/kg, *p* = 0.034) were statistically significantly negatively associated with total fat mass. RMSSD (− 0.790 ms/1%, *p* = 0.007) and pNN50 (− 0.296%/1%, *p* = 0.019) were statistically significantly negatively associated with percent body fat. No HRV frequency domain measures were statistically significantly associated with total fat mass or percent body fat.

**Table 3:** Associations between Total Body Composition Measures (total fat mass and percent body fat) and HRV Measures among 8–17-year-olds, adjusted for Sex and Tanner stage

	**Total Fat Mass (kg)**			**Percent Body Fat (%)**		
	**Coefficient ** **(95% CI)**	***p*-value**	**Adjusted R**^**2**^	**Coefficient** **(95% CI)**	***p*-value**	**Adjusted R**^**2**^
RMSSD (ms)	− 0.558 (− 0.941, − 0.175)	0.004^†^	0.023	− 0.790 (− 1.367, − 0.213)	0.007^†^	0.020
pNN50 (%)	− 0.178 (− 0.342, − 0.014)	0.034^†^	0.007	− 0.296 (− 0.542, − 0.049)	0.019^†^	0.010
LF Power (NU)	0.093 (− 0.036, 0.221)	0.157	-0.001	0.161 (− 0.031, 0.354)	0.100	0.001
HF Power (NU)	− 0.093 (− 0.221, 0.036)	0.157	-0.001	− 0.161 (− 0.354, 0.031)	0.100	0.001
LF:HF*	0.370 (− 0.232, 0.975)	0.228	-0.003	0.628 (− 0.293, 1.558)	0.181	0.001

Abbreviations: HF, high frequency; LF, low frequency; LF:HF, low frequency to high frequency ratio; NU, normalized units; pNN50, percentage of NN intervals greater than 50 ms; RMSSD, root mean square of successive differences

^*^Natural logarithm transformed; coefficient represents percent change per unit

Table 4 shows results from linear regression models evaluating associations between regional body composition and HRV measures, adjusted for sex and Tanner stage. RMSSD was statistically significantly negatively associated with trunk FM (− 1.024 ms/kg, *p* = 0.004), appendicular fat mass (− 1.152 ms/kg, *p* = 0.008), android FM (− 0.005 ms/g, *p* = 0.006), and gynoid FM (− 0.003 ms/g, *p* = 0.005). pNN50 was statistically significantly negatively associated with trunk FM (− 0.344%/kg, *p* = 0.022), android FM (− 0.002 ms/g, *p* = 0.028), and gynoid FM (− 0.001 ms/g, *p* = 0.034). RMSSD (− 0.013 ms/g, *p* = 0.019) and pNN50 (− 0.005%/g, *p* = 0.038) were statistically significantly negatively associated with VAT. RMSSD (− 0.007 ms/g, *p* = 0.004) and pNN50 (− 0.003%/g, *p* = 0.017) were statistically significantly negatively associated with SAAT. LF power (0.004 NU/g, *p* = 0.028), HF power (− 0.004 NU/g, *p* = 0.028), and the natural logarithm of LF:HF ratio (0.019%/g, *p* = 0.033) were statistically significantly associated with VAT.

**Table 4:** Associations between Regional Adiposity and Heart Rate Variability Measures among 8–17-year-olds, adjusted for Sex and Tanner stage

	**Trunk Fat Mass (kg)**			**Appendicular Fat Mass (kg)**		
	**Coefficient** **(95% CI)**	***p*-value**	**Adjusted R**^**2**^	**Coefficient** **(95% CI)**	***p*-value**	**Adjusted R**^**2**^
RMSSD (ms)	− 1.024 (− 1.709, − 0.338)	0.004^†^	0.024	− 1.152 (− 1.995, − 0.310)	0.008^†^	0.020
pNN50 (%)	− 0.344 (− 0.637, − 0.051)	0.022^†^	0.009	− 0.340 (− 0.701, 0.021)	0.065	0.003
LF Power (NU)	0.192 (− 0.037, 0.421)	0.100	0.001	0.157 (− 0.125, 0.439)	0.274	− 0.003
HF Power (NU)	− 0.192 − 0.421, 0.037	0.100	0.001	− 0.157 (− 0.439, 0.125)	0.274	− 0.003
LF:HF*	0.812 (− 0.266, 1.901)	0.140	− 2.742e^−4^	0.565 (− 0.755, 1.902)	0.402	− 0.005

	**Android Fat Mass (g)**			**Gynoid Fat Mass (g)**		
	**Coefficient** **(95% CI)**	***p*-value**	**Adjusted R**^**2**^	**Coefficient** **(95% CI)**	***p*-value**	**Adjusted R**^**2**^
RMSSD (ms)	− 0.005 (0.008, − 0.001)	0.006^†^	0.022	− 0.003 (− 0.005, − 0.001)	0.005^†^	0.023
pNN50 (%)	− 0.002 (− 0.003, − 1.791e^−4^)	0.028^†^	0.008	− 0.001 (− 0.002, − 7.714e^−5^)	0.034^†^	0.007
LF Power (NU)	0.001 (− 2.067e^−4^, 0.002)	0.106	0.001	3.118e^−4^ (− 4.012e^−4^, 0.001)	0.390	− 0.005
HF Power (NU)	− 0.001 (− 0.002, 2.067e^−4^)	0.106	0.001	− 3.118e^−4^ (− 0.001, 4.012e^−4^)	0.390	− 0.005
LF:HF*	0.004 (− 0.001, 0.010)	0.152	− 0.001	0.001 (− 0.002, 0.005)	0.512	− 0.006

	**VAT (g)**			**SAAT (g)**		
	**Coefficient** **(95% CI)**	***p*-value**	**Adjusted R**^**2**^	**Coefficient** **(95% CI)**	***p*-value**	**Adjusted R**^**2**^
RMSSD (ms)	− 0.013 (− 0.025, − 0.002)	0.019^†^	0.014	− 0.007 (− 0.013, − 0.002)	0.004^†^	0.023
pNN50 (%)	− 0.005 (− 0.010, − 2.728e^−4^)	0.038^†^	0.006	− 0.003 (− 0.005, − 4.851e^−4^)	0.017^†^	0.009
LF Power (NU)	0.004 (4.533e^−4^, 0.008)	0.028^†^	0.005	0.002 (− 1.123e^−4^, 0.003)	0.067	− 5.534e^−7^
HF Power (NU)	− 0.004 (− 0.008, − 4.533e^−4^)	0.028^†^	0.005	− 0.002 (− 0.003, 1.123e^−4^)	0.067	− 5.534e^−7^
LF:HF*	0.019 (0.002, 0.036)	0.033^†^	0.003	0.007 (− 0.001, 0.015)	0.089	− 0.003

Abbreviations: HF, high frequency; LF, low frequency; LF:HF, low frequency to high frequency ratio; NU, normalized units; pNN50, percentage of NN intervals greater than 50 ms; RMSSD, root mean square of successive differences; SAAT, subcutaneous abdominal adipose tissue; VAT, visceral adipose tissue

^*^Natural logarithm transformed; coefficient represents percent change per unit

### Relationships between Heart Rate Variability, Gonadotropins, and Sex Hormones

A total of 110 children and adolescents were included in this secondary sub-analysis (54% female, mean age 12.2 ± 2.6 years old; see Supplementary Table 1 for baseline characteristics for this subset). Within this subset the majority (59%) were pubertal, predominately identified as non-Hispanic White (75%) and were categorized as having obesity (53%). After stratifying by sex and adjusting for Tanner stage category, no measures of HRV were statistically significantly associated with LH, FSH, estrogen, or testosterone (see Tables 5 and 6).

**Table 5:** Associations between Gonadotropins and Sex Hormone Measures with Heart Rate Variability Measures among 8–17 Year Old’s, Adjusted for Tanner stage category in Females

	**FSH**			**LH**		
	**Coefficient** **(95% CI)**	***p*-value**	**Adjusted R**^**2**^	**Coefficient** **(95% CI)**	***p*-value**	**Adjusted R**^**2**^
RMSSD (ms)	− 4.015 (− 20.767, 13.195)	0.632	− 0.034	1.755 (− 4.761, 8.271)	0.590	− 0.034
pNN50 (%)	0.090 (− 6.417, 6.597)	0.978	− 0.046	0.809 (− 1.991, 3.609)	0.563	− 0.055
LF Power (NU)	− 0.965 (− 5.258, 3.329)	0.653	− 0.056	− 0.414 (− 2.272, 1.444)	0.655	− 0.045
HF Power (NU)	0.965 (− 3.329, 5.258)	0.653	− 0.056	0.414 (− 1.444, 2.272)	0.655	− 0.045
LF:HF*	− 5.611 (− 22.324, 14.697)	0.554	− 0.055	− 2.257 (− 10.395, 6.619)	0.599	− 0.049

	**Testosterone**			**Estradiol**		
	**Coefficient** **(95% CI)**	***p*-value**	**Adjusted R**^**2**^	**Coefficient** **(95% CI)**	***p*-value**	**Adjusted R**^**2**^
RMSSD (ms)	− 24.710 (− 64.554, 15.129)	0.217	0.026	− 0.033 (− 0.379, 0.313)	0.850	− 0.042
pNN50 (%)	− 12.460 (− 29.978, 5.058)	0.158	− 0.018	0.023 (− 0.115, 0.161)	0.739	− 0.047
LF Power (NU)	− 1.355 (− 13.981, 11.272)	0.829	− 0.069	0.001 (− 0.094, 0.095)	0.989	− 0.051
HF Power (NU)	1.355 (− 11.272, 13.981)	0.829	− 0.069	− 0.001 (− 0.095, 0.094)	0.989	− 0.051
LF:HF*	− 4.421 (− 46.461, 70.630)	0.889	− 0.050	0.002 (− 0.438, 0.435)	0.992	− 0.053

Abbreviations: FSH, follicle stimulating hormone; HF, high frequency; LF, low frequency; LF:HF, low frequency to high frequency ratio; LH, luteinizing hormone; NU, normalized units; pNN50, percentage of NN intervals greater than 50 ms; RMSSD, root mean square of successive differences

^*^Natural logarithm transformed; coefficient represents percent change per unit

**Table 6:** Associations between Gonadotropins and Sex Hormone Measures with Heart Rate Variability Measures among 8–17 Year Old’s, Adjusted for Tanner stage category in Males

	**FSH**			**LH**		
	**Coefficient** **(95% CI)**	***p*-value**	**Adjusted R**^**2**^	**Coefficient** **(95% CI)**	***p*-value**	**Adjusted R**^**2**^
RMSSD (ms)	10.97 (− 19.729, 41.677)	0.473	0.034	4.093 (− 15.262, 23.448)	0.670	0.004
pNN50 (%)	4.358 (− 8.370, 17.086)	0.492	− 0.036	0.882 (− 6.908, 8.671)	0.820	− 0.062
LF Power (NU)	− 3.364 (− 13.622, 6.894)	0.510	− 0.070	− 3.225 (− 9.572, 3.122)	0.309	− 0.053
HF Power (NU)	3.364 (− 6.894, 13.622)	0.510	− 0.070	3.225 (− 3.122, 9.572)	0.309	− 0.053
LF:HF*	− 16.474 (− 48.853, 36.405)	0.462	− 0.067	− 15.143 (− 37.200, 14.666)	0.276	− 0.046

	**Testosterone**			**Estradiol**		
	**Coefficient** **(95% CI)**	***p*-value**	**Adjusted R**^**2**^	**Coefficient** **(95% CI)**	***p*-value**	**Adjusted R**^**2**^
RMSSD (ms)	6.737 (− 12.298, 25.773)	0.474	0.026	− 0.527 (− 2.130, 1.077)	0.510	− 0.023
pNN50 (%)	− 0.402 (− 8.257, 7.543)	0.917	− 0.075	− 0.108 (− 0.779, 0.563)	0.746	− 0.046
LF Power (NU)	− 2.476 (− 8.554, 3.603)	0.411	− 0.008	− 0.026 (− 0.557, 0.505)	0.921	− 0.068
HF Power (NU)	2.476 (− 3.603, 8.554)	0.411	− 0.008	0.026 (− 0.505, 0.557)	0.921	− 0.068
LF:HF*	− 11.706 (− 34.390, 18.823)	0.398	− 0.024	− 0.102 (− 2.628, 2.490)	0.936	− 0.070

Abbreviations: FSH, follicle stimulating hormone; HF, high frequency; LF, low frequency; LF:HF, low frequency to high frequency ratio; LH, luteinizing hormone; NU, normalized units; pNN50, percentage of NN intervals greater than 50 ms; RMSSD, root mean square of successive differences

^*^Natural logarithm transformed; coefficient represents percent change per unit

## Discussion

In this study, we sought to determine associations between adiposity and HRV measures, and to assess associations between gonadotropins as well as sex hormones, and HRV measures, in a cohort of 8–17-year-old children and adolescents with BMIs ranging from normal weight to obese. Overall, we found that increased adiposity was associated with worsened HRV. While previous research examining HRV and body composition in youth has been limited, here we offer a more in-depth analysis including total and regional body composition measures alongside HRV outcomes. To our knowledge, this is the first study to examine associations between HRV and both total and regional adiposity using linear regression analysis in a large cohort consisting of both children and adolescents.

More specifically, our study aimed to investigate associations between specific levels of adiposity and measures of HRV, and to understand the significance of these effects. We found that RMSSD was statistically significantly negatively associated with all measures of FM (total, trunk, appendicular, android, gynoid, visceral, and subcutaneous abdominal), and that pNN50 was negatively associated with all measures of FM with the exception of appendicular adipose tissue. RMSSD and pNN50, both time domain metrics, measure variability of time between heart beats. Combined, these findings show that with increased adiposity there is decreasing variability between heartbeats, suggesting that increased adiposity may be detrimental to cANS functioning. We also found that LF power, and LF to HF ratio was statistically significantly positively associated with VAT but not with SAAT, while HF power was negatively associated with only VAT. As LF power has been linked to sympathetic activity, and HF power linked to vagal tone in adults [36], our results suggest that central adiposity, but not peripheral adiposity, show an association with decreased vagal tone and sympathetic dominance [37].

Research by Gutin et al. [25] explored relationships between adiposity and HRV measures among 168 adolescents (14–18 years old) and found a negative association between VAT and RMSSD, along with positive associations between VAT and LF:HF ratio, and between SAAT and LF:HF ratio. We found similar significant associations after adjusting for sex and Tanner stage; specifically, that VAT was negatively associated with RMSSD and HF power, and positively associated with LF power. While we did not see associations between SAAT and frequency domain measures, the difference between previous results and our results may be due to differences in how SAAT was determined between studies.

Another study by Kaufman et al. [26] examined correlations between HRV and DXA-derived adiposity measures among 36 children (10–13 years old) and found no significant correlations between HRV and total or trunk percent fat after adjusting for age, C-reactive protein, Tanner stage, and insulin. In contrast, we found statistically significant associations between nearly all adiposity measures assessed and HRV time domain measures after adjusting for sex and Tanner stage. Our findings suggest that as adiposity increases, there may be an associated reduction in vagal tone, which has been associated with metabolic syndrome risk factors and future cardiovascular risk in youth [38, 39], and cardiovascular disease and type 2 diabetes in adults [40, 41]. Of note, our study differed from Kaufman et al. [26] in several important ways, including model covariates adjusted for, the age range (10–13 versus 8–17 years old as in our study), and analyses performed. Specifically, while Kaufman et al. [26] used correlation measures, we used linear regression modeling.

Additional studies have examined HRV alongside adiposity, also in pre-pubertal cohorts [27, 28]. One particular study examining a cohort of 575 children from Guadeloupe found, using restricted cubic splines models, no significant associations between BMI z-score and HRV under calm conditions [27], contrasting with the current study results. Discrepancies in results may be attributed to the previous study utilizing BMI z-scores as an adiposity indicator [27]. In another study [28] of 50 Caucasian children (6–10 years), HRV was examined in relation to total fat mass and trunk fat mass determined via DXA. Partial correlations adjusting for age, Tanner stage, and moderate to vigorous physical activity levels found associations between both adiposity measures and natural log transformed frequency domain HRV measures, but not with time domain measure [28]. Differences in outcomes could again be due to differences in sample size, covariates included, and statistical approach.

Total fat is well understood to have an impact on the physiologic functioning of individuals, including children and adolescents. Children with higher fat mass have been shown to have increased metabolic syndrome risk score [42], and adolescents with excess adiposity, despite having normal weight, are at an increased risk of developing cardiometabolic risk factors and diseases [43]. While previous studies are inconsistent in demonstrated associations between total body adiposity and HRV, the current study found total fat mass and percent body fat to be significantly associated with decreased time domain measures of HRV. Further research is needed to better understand the potential impact of total body adiposity on cardiac autonomic function.

It is well known that fat deposition in different areas of the body can elicit differential physiological responses. For example, studies have found that VAT shows stronger associations with adverse cardiovascular risk compared to other adiposity measures in adults [44, 45] and that central adiposity (measured via waist circumference) is associated with poorer ANS function in younger adults, while BMI (used as a surrogate measure of total adiposity) has not been [24]. Previous literature has also demonstrated that abdominal adiposity, compared to peripheral adiposity, has a stronger association with increased cardio-metabolic risk factors in children and adolescents [46–48]. To our knowledge no studies have explored if central adiposity shows stronger associations compared to peripheral when specifically examining associations with HRV in a cohort of children and adolescents.

We also explored whether central, versus peripheral, adiposity differs in its effect on HRV, and found that both VAT and SAAT were statistically significantly negatively associated with both RMSSD and pNN50. Adjusted R^2^ values show a better model fit for SAAT; however, this may also be explained by the much greater SAAT levels compared to VAT. The regression coefficient shows that per gram of adipose tissue, VAT has a greater negative association with RMSSD and pNN50, and VAT was significantly associated with worsened frequency domain measures. These results suggest that VAT may have a broader association with HRV, as it was negatively associated with all measures of HRV. Similarly, both android and gynoid fat mass showed significant negative associations with RMSSD and pNN50 with similar adjusted R^2^ values, though per gram android fat showed a greater negative association with RMSSD and pNN50. Decreased RMSSD and pNN50 both represent decreased variability between heartbeats, which may be indicative of reduced cardiac autonomic nervous system flexibility and responsiveness in children [49]. Given these findings, it may be that those with a disposition to store fat in the android region have greater decreases in HRV and may be at greater risk of sympathetic dominance, which has been shown to be linked to proarrhythmic conditions and chronic heart failure in adults [50–53].

To further our understanding of central versus peripheral adiposity in relation to HRV, we examined if trunk and appendicular adiposity associations with HRV differed and found that appendicular fat mass showed a greater per kg negative association with RMSSD compared to trunk fat. However, appendicular fat mass was not associated with pNN50, while trunk fat mass was statistically significantly negatively associated. These findings suggest that, though appendicular fat showed a slightly larger effect on RMSSD, trunk fat may have a broader association with HRV as it was negatively associated with both RMSSD and pNN50.

Pediatric obesity, often a prelude to adult obesity, is a major public health concern in the United States and worldwide [54]. Obesity in adulthood is associated with the development of numerous adverse health sequelae including type 2 diabetes mellitus, cardiovascular disease, chronic inflammation, and hyperinsulinemia, among others [55]. These comorbidities, also seen in children and adolescents with obesity, can be severe and may have a cascading effect leading to autonomic dysfunction [56–59]. The development of obesity can lead to a phenotypic switch in white adipose tissue, which secretes systemic adipokines and cytokines [16, 60]. This switch may cause inflamed, dysfunctional adipocytes that secret systemic pro-inflammatory cytokines that can then disrupt normal adipose tissue [16, 60]. Previous research has pointed towards associations between inflammation and RMSSD, which is one possible mechanism that could underlie our findings which showed that nearly all measures of adipose tissue were statistically significantly negatively associated with RMSSD and pNN50.

Previous studies have also shown lower HRV to be associated with adverse cardiac events, stroke, and mortality independent of resting heart rate in adults [61] and other standard cardiovascular risk factors [61, 62]. Literature and existing data have also supported the use of HRV as a preclinical maker for several clinical outcomes, including atrial fibrillation and stroke [8, 9, 61, 63–65]. Adiposity and obesity are widely known to be associated with increased cardiovascular mortality and morbidity, and one mechanism for this increased risk may be via adiposity’s effect on cANS functioning [66]. Our study provides evidence for specific associations between adiposity and worsened cardiac autonomic nervous system function in children and adolescents.

A strength of our study is our large sample size of children and adolescents, generally larger than similar studies [25, 26]. Additionally, body composition was determined using DXA, which allowed for the determination of both total and regional body composition measures. Our study must also be interpreted in the context of limitations. For one, as our population was predominately non-Hispanic White, results may not be generalizable to other racial/ethnic groups, including those who may already be at higher risk of cardiovascular and metabolic diseases, including non-Hispanic Black and Hispanic populations [67]. Another limitation is that, in the present study, Tanner staging was performed using only visual inspection of pubic hair, which is a marker of adrenarche rather than puberty (breast development for females and testicular size for males). Adrenarche and puberty are generally highly correlated, however not always, particularly in youth with obesity [68]. Additionally, as this was a cross-sectional study, we could only determine associations rather than causation. Moreover, we were not able to determine the role that socioeconomic status (SES) may play in these relations given substantial missing-ness of such variables in the dataset used. Finally, measures of physical fitness/activity were not accounted for within this analysis, thus we were not able to determine if these associations exist independent of these variables. In conclusion, our results provide evidence for a potential negative influence of excess adipose tissue on HRV measures in children and adolescents of varying weight statuses ranging from normal weight to obesity. Our findings suggest that increasing adiposity may negatively affect cardiac autonomic nervous system functioning. Future studies, particularly prospective cohort trials, are needed to confirm these relationships and to determine potential interventions aimed at risk reduction.

## Supplementary Information

Below is the link to the electronic supplementary material.

Supplementary file1 (DOCX 15 kb)

## Data Availability

No datasets were generated or analysed during the current study.
