# Supplementary material for: Relationships Between Adiposity Measures and Heart Rate Variability in Children and Adolescents
Source: Pediatr Cardiol. 2025 Jul 10;47(4):1490–500. doi: 10.1007/s00246-025-03924-3 (PMC12945889; doi:10.1007/s00246-025-03924-3)
Supplement: Supplementary file 1 — Supplementary file1 (DOCX 15 kb) [file 246_2025_3924_MOESM1_ESM.docx]

**Supplementary Table 1**: Baseline Characteristics of 110 Children and Adolescents (8-17 years) with available Gonadotropin and Sex Hormone Data.

|  | **Value** |
| --- | --- |
| **Age (years), mean (SD)** | 12.2 (2.6) |
| **Female, n (%)** | 59 (54) |
| **Race/Ethnicity, n (%)** |  |
| White (non-Hispanic) | 83 (75) |
| Black (non-Hispanic) | 7 (6) |
| Hispanic/Latino | 14 (13) |
| Asian | 2 (2) |
| Multi-Racial | 3 (3) |
| Other | 1 (1) |
| **Tanner Stage, n (%)** |  |
| Stage I | 32 (29) |
| Stage II-IV | 65 (59) |
| Stage V | 13 (12) |
| **Weight Status, n (%)** |  |
| Normal Weight | 43 (39) |
| Overweight | 9 (8) |
| Obesity | 58 (53) |
